# Rehospitalization Burden Profiles After Traumatic Spinal Cord Injury: A Data-Driven Latent Class Analysis of the SCIMS Public-Use Database

**DOI:** 10.3390/jcm15134890

**Published:** 2026-06-23

**Authors:** Andrea Calderone, Maria Pia Onesta, Laura Simoncini, Antonino Nunnari, Fabrizio Sottile, Angelo Quartarone, Rocco Salvatore Calabrò

**Affiliations:** 1 IRCCS Centro Neurolesi Bonino-Pulejo, 98124 Messina, Italy; 2 Spinal Unit, Montecatone Rehabilitation Institute, 40026 Imola, Italy; 3 Unipolar Spinal Unit, AOE Cannizzaro, 95126 Catania, Italy; laura.simoncini@montecatone.com

**Keywords:** spinal cord injury, rehospitalization, latent class analysis, burden profiles, participation, SCIMS, pressure ulcer, urinary tract infection

## Abstract

**Background/Objectives:** Rehospitalization after traumatic spinal cord injury (SCI) is common, but binary or count summaries may obscure heterogeneity in timing, recurrence, frequency, and duration. We aimed to identify clinically interpretable rehospitalization burden profiles in the SCIMS 2021ARPublic dataset and examine descriptive associations with clinical correlates and participation outcomes. **Methods:** We analyzed Form I, Form II, and Record Status public-use files. Among 29,310 individuals with at least one non-lost follow-up interview, 28,745 with at least one non-missing rehospitalization indicator entered latent class analysis. Four prespecified indicators captured early, recurrent, frequent, and prolonged rehospitalization. Candidate two- through six-class models were compared using AIC, BIC, entropy, class size, posterior probabilities, and interpretability. Pairwise adjusted logistic models examined candidate clinical correlates in 10,407 participants with complete 2016+ follow-up data. Adjusted linear models examined CHART participation domains in 20,766–20,949 participants. **Results:** A four-profile solution was retained: low rehospitalization burden (59.8%), early/prolonged rehospitalization (18.9%), frequent/prolonged rehospitalization (7.7%), and high recurrent/frequent/prolonged burden (13.6%). UTI and pressure ulcer history showed the most consistent associations with burdened profiles. Severe pain and frequent sleep problems were associated with selected heavier-burden profiles, while depressive symptoms showed smaller and less precise associations. Sensitivity analyses supported structural stability while highlighting observation-time bias and classification uncertainty inherent to wave-based public-use data. Compared with the low-burden profile, burden profiles showed lower CHART scores, especially for mobility and occupation. **Conclusions:** Rehospitalization after traumatic SCI is heterogeneous. These utilization burden profiles summarize distinct observed patterns but require prospective validation before use in risk stratification or follow-up planning.

## 1. Introduction

Rehospitalization after traumatic spinal cord injury (SCI) remains common and clinically important, but the burden is unevenly distributed. Meta-analytic evidence indicates that early readmission after traumatic SCI is frequent [1]. Population-based work suggests that rehospitalization remains relevant beyond the immediate post-discharge period [2], and longer-term analyses show that recurrent admissions can continue over several post-injury years [3]. Earlier multicenter work also showed that rehospitalization reflects diverse etiologies rather than a single dominant mechanism [4]. This heterogeneity matters because SCI is associated with a substantial economic burden, and rehospitalization can intensify downstream costs [5]. Analyses from the Spinal Cord Injury Model Systems (SCIMS) National Database further underline that people living with traumatic SCI often carry complex and overlapping health needs over time [6].

Secondary conditions remain central to post-discharge neurorehabilitation pathways. Urinary tract infection (UTI) is one of the most common complications after SCI and is shaped by neurogenic bladder management, catheter practices, diagnostic uncertainty, and altered symptom expression [7,8]. Recurrent UTI may reflect broader interactions among bladder physiology, device use, antimicrobial exposure, and access to timely management [9]. Skin complications are similarly important, and SCI-specific guidance emphasizes systematic pressure injury prevention and surveillance [10]. Contemporary evidence syntheses indicate that pressure injuries remain frequent and recurrent lesions are influenced by multiple interacting risks [11,12].

Symptom burden may also accompany heavier utilization. Chronic pain is highly prevalent after SCI and remains difficult to manage [13], and longitudinal work suggests that pain trajectories relate to psychosocial resources and mental health [14]. Sleep disturbance and depressive symptoms are also common and may complicate recovery, self-management, and participation [15,16,17,18]. These domains do not establish a causal pathway to rehospitalization, but they support examining symptoms and complication burden as clinical correlates when describing utilization profiles.

Many secondary analyses reduce rehospitalization to a binary event, a single annual count, or total inpatient days. Such summaries are useful for surveillance and prediction, yet they may miss differences in timing, recurrence across follow-up waves, within-wave frequency, and duration. An individual with one early readmission may differ meaningfully from an individual with repeated admissions across several follow-up waves, even if both are classified as rehospitalized. Latent class analysis (LCA) offers a pragmatic descriptive approach because it identifies subgroups defined by shared probability patterns rather than by a single arbitrary threshold [19,20,21]. In this setting, LCA is not used to infer biological subtypes or causal mechanisms, but to summarize observed utilization burden in a clinically readable way.

The SCIMS Public Use Dataset is well-suited to this question. The SCIMS National Database is one of the most established longitudinal resources for traumatic SCI research and has documented representativeness relative to the broader United States inpatient rehabilitation population with new traumatic SCI [22]. Its follow-up structure enables the study of rehospitalization over multiple post-injury intervals with standardized data collection. Utilization burden is clinically relevant when it aligns with participation, a central rehabilitation outcome [23,24,25,26,27]. The present study therefore aimed to identify rehospitalization burden profiles after traumatic SCI using the SCIMS Public Use Dataset and to examine their descriptive associations with candidate clinical correlates and long-term participation outcomes. We expected that profile-based summaries would communicate heterogeneity in timing, recurrence, frequency, and duration while remaining complementary to simpler count/day measures.

## 2. Materials and Methods

### 2.1. Study Design, Reporting Framework, and Data Source

This study was a secondary analysis of the SCIMS Public Use Dataset, version 2021ARPublic. The analysis used all three publicly available files released with this version, namely Form I, Form II, and Record Status. Form I contains baseline and discharge information from the index system hospitalization. Form II contains repeated follow-up interviews conducted at post-injury anniversary years. Record Status contains follow-up tracking and vital status information. Because this was an observational analysis of routinely collected de-identified data, the Methods section was structured with attention to the Strengthening the Reporting of Observational Studies in Epidemiology and the REporting of studies Conducted using Observational Routinely collected health Data recommendations [28,29]. The SCIMS database was selected because it is an established longitudinal resource for traumatic SCI research in the United States, with documented representativeness and contemporary epidemiologic profiling that support cautious generalization within the limits of the public-use release [30,31].

The 2021ARPublic release contains 35,675 Form I records, 131,217 Form II records, and 35,675 Record Status records. Form II corresponds to 32,541 unique persons. Of the 131,217 Form II observations, 58,377 are coded as lost to follow-up, leaving 72,840 non-lost interviews and 29,310 persons with at least one useful follow-up record. The public-use release is de-identified. Dates of injury, admission, discharge, interview, annual examination, vital status, and death are retained only at a yearly resolution. Age at injury is released in a grouped public-use form rather than as an exact age. These privacy protections have direct analytic implications. Exact time-to-event modeling is not possible from the public-use files, and analytic time must therefore be represented primarily by anniversary-wave structure rather than by calendar dates or exact elapsed days.

### 2.2. Cohort Assembly, Linkage Keys, Follow-Up Structure, and Analytic Time Scale

Records were linked using the public-use identifier structure supplied by the National Spinal Cord Injury Statistical Center. The unique identification key for Form I and Record Status is UniID. The identifying key for Form II is the combination of UniID and post-injury anniversary year, recorded as BYear. Record linkage was first used to assemble a participant-level cohort anchored on Form I. Form II interviews were then merged with the baseline cohort using UniID and BYear, while Record Status was used for cohort accounting and consistency checks related to follow-up eligibility and vital status.

Not every Form I observation has a corresponding Form II interview in the public-use release. This is expected under SCIMS follow-up rules because some participants were not yet due for the first anniversary interview by the close of data collection, because follow-up is not required for those with neurologic recovery at discharge from the initial hospitalization, and because some participants died before the first anniversary. In addition, SCIMS policy does not require submission of a Year 1 Form II while the initial hospitalization is still ongoing. In that setting, the first post-discharge interview may occur later and may function operationally as the first follow-up record. For the present analysis, all Form II records coded as lost to follow-up were treated as not observed for outcome ascertainment and were excluded from the construction of rehospitalization and participation measures. Lost follow-up was identified by Category of Follow-up Care equal to 5 (BFolUpCt = 5). All remaining non-lost Form II records were eligible, regardless of whether they reflected direct system care or interview-only follow-up.

**Figure 1:**
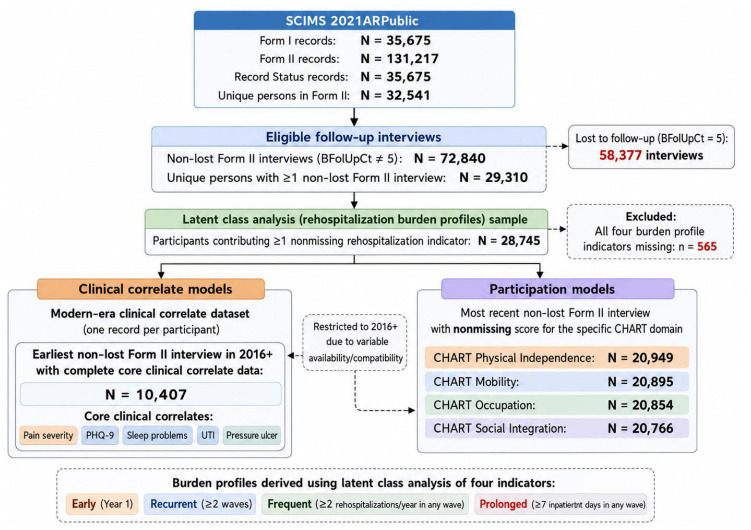
Cohort assembly and analytic samples for SCIMS 2021ARPublic. The profile cohort included 28,745 participants; clinical-correlate models used 10,407 participants; and CHART models used domain-specific samples of 20,949, 20,895, 20,854, and 20,766 participants.

Analytic time was represented using BYear as a discrete post-injury wave. The SCIMS public-use release includes post-injury years 1, 5, 10, 15, 20, 25, 30, 35, 40, 45, and 50, with additional years permitted in the database if submitted. For the rehospitalization burden profile analysis, all available non-lost follow-up waves were used rather than restricting the cohort to a small set of anniversary years. Because dates are retained only as years, the analysis was designed around wave-level patterns rather than exact event timing. Recurrent, frequent, and prolonged indicators were defined as ever positive across observed waves and therefore could be influenced by the amount of follow-up observed. We consequently summarized follow-up intensity by profile and conducted sensitivity analyses restricted to participants with at least two and at least three informative non-lost waves. Record Status was retained for cohort accounting and for consistency checks related to follow-up eligibility and vital status. The complete cohort assembly and derivation of the analysis-specific samples are summarized in Figure 1.

### 2.3. Measures and Operational Definitions

The primary analytic domain was rehospitalization burden, derived from Form II variables that summarize overnight hospital admissions occurring after discharge from the index system hospitalization. The key public-use variables were Number of Rehospitalizations During the Last 12 Months (BRhspNbr), Number of Days Rehospitalized During the Last 12 Months (BRhspDaT), and up to eight episode-specific rehospitalization reason variables (BRhspRs1-BRhspRs8). BRhspNbr captures planned and unplanned admissions in system and non-system hospitals. For Year 1 only, the reporting interval is the period from discharge from the initial system hospitalization to the interview date. For subsequent follow-ups, the reporting interval is the preceding 12 months. BRhspNbr is auto-calculated within the SCIMS data-collection system, as documented in the SCIMS Public Use Dataset, version 2021ARPublic, and is coded 0 for none; 1–6 for the exact number of admissions; 7 for more than six admissions; 8 for rehospitalized with an unknown number; and 9 for unknown. BRhspDaT is also auto-calculated within the same SCIMS 2021ARPublic data framework and is coded 0 for none, 1–887 for valid total inpatient days, 888 for rehospitalized with an unknown number of days, and 999 for unknown.

Four binary indicators were prespecified to define rehospitalization burden profiles in a clinically interpretable way. Early rehospitalization was defined as any rehospitalization recorded on the standard Year 1 Form II interview. A positive value of BRhspNbr was interpreted as evidence of rehospitalization, whereas BRhspNbr = 9 was treated as missing. Recurrent rehospitalization was defined as rehospitalization reported in at least two informative non-lost Form II waves. Participants with fewer than two informative waves were set to missing for this specific indicator, rather than forced into the nonrecurrent category. Frequent rehospitalization was defined as two or more admissions in any observed follow-up wave, with BRhspNbr codes 2–7 treated as positive and BRhspNbr = 8 treated as missing for thresholding because the count was unknown. Prolonged rehospitalization was defined as seven or more total inpatient days in any observed follow-up wave, with BRhspDaT = 888 and BRhspDaT = 999 treated as missing. These thresholds were selected as transparent operational definitions for clinical interpretation and were examined using stricter sensitivity cut points of at least three admissions and at least fourteen inpatient days.

Candidate clinical correlates were defined a priori from Form II to reflect symptom burden and common secondary complications relevant to neurorehabilitation pathways. Pain severity was measured using BPainSev, which records usual pain over the last four weeks on a 0–10 scale; values 0–10 were valid, and 99 was missing. For readability, pain severity was categorized as 0–3, 4–6, and 7–10. Depressive symptoms were measured using the Patient Health Questionnaire-9 severity score (BBPHQSDS), coded 0–27 with 99 representing unknown, declined, interview not done, or age younger than 18 years [32]. Clinically significant depressive symptoms were defined as PHQ-9 ≥ 10. Sleep problems were measured using BSleep and summarized as weekly/daily versus less frequent. Urinary tract infection requiring antibiotics in the last 12 months was captured by BUTI, and pressure ulcer in the last 12 months by BPrUlcer. Declined and unknown responses were treated as missing. Because variable availability changed across program eras, the clinical-correlate analysis was restricted to the earliest non-lost Form II interview in 2016 or later with complete data on all core correlates. These variables were treated as descriptive clinical correlates, not causal predictors, because they may have preceded, overlapped with, or followed rehospitalization events used for profile assignment.

Participation outcomes were defined using CHART domain totals available in Form II, namely CHART Physical Independence Total (BCHPITot), CHART Mobility Total (BCHMbTot), CHART Occupation Total (BCHOpTot), and CHART Social Integration Total (BCHSocIn) [33,34]. Each domain ranges from 0 to 100 in the public-use release, with 999 representing unknown or an interview not done. Outcomes were taken from the most recent non-lost follow-up interview with a valid score for the specific domain. Domain-specific regression models used available case denominators after excluding 999-coded values. Because universally accepted minimal clinically important differences for each CHART domain in this registry context are not established, differences were interpreted using pragmatic 0–100 scale anchors: <5 points as small, 5–9 points as potentially meaningful or moderate, and ≥10 points as large.

Baseline covariates were prespecified from Form I to preserve temporal precedence and address reviewer concerns regarding neurological and demographic factors. The core adjusted set included age at injury, sex, race, Hispanic origin, broad traumatic etiology, discharge neurological level region, and discharge American Spinal Injury Association Impairment Scale grade (AASAImDs), subject to data completeness and model stability. Discharge neurological category (ANCatDis) was evaluated but not retained in all pairwise models because of collinearity and instability with neurological level and AIS grade. Form II neurological measures were not used as primary repeated covariates because they are required only at the first anniversary and are optional thereafter in the public-use release, which would introduce nonuniform completeness by wave.

### 2.4. Rehospitalization Burden-Profile Derivation Strategy

Rehospitalization burden profiles were derived using latent class analysis applied to the four binary indicators described above. This approach was selected because it provides a probabilistic framework for identifying unobserved subgroups defined by shared patterns of early, recurrent, frequent, and prolonged rehospitalization without imposing a single threshold definition of burden [35,36]. The model assumed local conditional independence of the four indicators within a profile. This was treated as a working model assumption rather than as a verified property of the data, and bivariate residual diagnostics were examined in sensitivity analyses. Missing indicator values were accommodated through full-information maximum likelihood so that participants contributed all available observed indicator information to profile estimation.

Candidate models with two through six profiles were estimated. Model selection was based on AIC, BIC, entropy, class size, average posterior probabilities, and clinical interpretability. A four-profile solution was retained because it had the lowest AIC and BIC and avoided the small, less stable additional partitions observed in more complex solutions. The profiles were labeled descriptively according to their dominant probability patterns as low rehospitalization burden, early/prolonged rehospitalization, frequent/prolonged rehospitalization, and high recurrent/frequent/prolonged burden. These labels summarize observed utilization profiles and do not imply biological subtypes, causal mechanisms, or latent disease states. Downstream models used modal profile assignment based on each participant’s highest posterior probability. Classification uncertainty was addressed through posterior-probability reporting and sensitivity analyses, excluding lower-certainty assignments.

### 2.5. Statistical Analysis Plan

The overall analytic strategy was designed to remain statistically straightforward and clinically interpretable. Baseline and follow-up characteristics were summarized overall and by burden profile. Continuous variables were described using mean and standard deviation or median and interquartile range as appropriate to distributional shape. Categorical variables were summarized using counts and percentages. Frequency tables and range checks were used to verify that all recoding decisions were consistent with the SCIMS data dictionary before modeling.

Associations between candidate clinical correlates and profile membership were analyzed using pairwise multivariable logistic regression, with the low rehospitalization burden profile as reference. The clinical-correlate dataset included one eligible harmonized interview per participant, defined as the earliest non-lost interview conducted in 2016 or later with complete core correlate information. Models were adjusted for the prespecified Form I covariates described above. Robust (sandwich) standard errors were used as a variance estimator to reduce sensitivity to heteroscedasticity and modest distributional misspecification, while recognizing that they do not correct an incorrect mean structure or establish causality [37]. Effect estimates were reported with 95% confidence intervals and interpreted as descriptive associations rather than causal effects.

Associations between profile membership and participation outcomes were estimated using linear regression with robust standard errors for each CHART domain separately. Each domain was modeled on the most recent non-lost follow-up with a valid score for that specific outcome. Sensitivity models additionally adjusted for CHART measurement BYear, number of non-lost interviews, and maximum observed BYear to examine whether measurement-time differences influenced the estimates. To address the added-value question, CHART models were also compared across baseline-only specifications, cumulative rehospitalization count/day plus exposure specifications, profile-only specifications, and combined count/day plus profile specifications.

Rehospitalization reasons from BRhspRs1-BRhspRs8 were analyzed descriptively. Non-missing reason positions were stacked to the event level, while code 88, indicating no rehospitalization, and code 99, indicating unknown reason, were excluded from descriptive cause summaries. These analyses were intended to contextualize the burden profiles rather than to define them. Formal recurrent-event survival models were not used because the public-use release does not retain exact hospital admission and discharge dates, and the rehospitalization variables summarize annual windows rather than gap times between events.

Several prespecified sensitivity analyses were used to assess robustness while keeping the overall strategy interpretable. First, profile derivation was repeated using stricter burden thresholds of three or more rehospitalizations in any wave and fourteen or more inpatient days in any wave. Second, LCA was repeated after restricting the cohort to participants with at least two and at least three informative non-lost follow-up waves. Third, key regression analyses were repeated after excluding participants with maximum posterior profile probabilities below 0.70 and below 0.80. Fourth, threshold-based models were re-estimated after excluding observations with BRhspNbr coded as rehospitalized but with the number unknown. Fifth, bivariate residual diagnostics were examined to evaluate residual conditional dependence among class-defining indicators.

### 2.6. Missing Data, Loss to Follow-Up, and Coding Conventions

Missingness in the SCIMS public-use release arises from multiple mechanisms and was handled with explicit distinction between structural missingness and item-level nonresponse. Structural missingness included interviews coded as lost to follow-up, variables that were not yet in the database during earlier program eras, variables retired and later reinstated, variables not required for specific waves, and values blank by design because BFolUpCt = 5. Item-level nonresponse included dictionary-defined declined, not applicable, or unknown responses. In the public-use documentation, codes 7, 8, and 9 often correspond to declined, not applicable, and unknown, although variable-specific coding schemes differ and were therefore applied according to each variable’s dictionary definition rather than by a single universal rule.

For rehospitalization measures, BRhspNbr = 8 was treated as positive for the binary indicator of any rehospitalization but as missing for the frequency threshold because the exact count is unknown. BRhspNbr = 9 was treated as missing. BRhspDaT = 888 and BRhspDaT = 999 were treated as missing for prolonged-stay thresholding. For clinical-correlate variables, BPainSev = 99, BBPHQSDS = 99, BSleep = 9, BUTI codes indicating declined or unknown, and BPrUlcer codes indicating declined or unknown were all treated as missing. For CHART outcomes, 999-coded values were treated as missing. Public-use blanks were not assumed to be equivalent to random omission. When a blank reflected a lost interview or a wave where a measure was not collected by design, that value was treated as structurally unavailable rather than simply absent.

No single-value imputation was used for primary regression analyses. Clinical-correlate and CHART models were estimated in model-specific complete-case samples, and denominators were allowed to vary according to the availability of the relevant outcome, exposure, and covariates. The latent class models used all available indicator information under a working missing-at-random assumption embedded in the full-information likelihood, but this assumption was not treated as proven. Because the main sources of missingness include program-era changes and lost follow-up, we favored design-based restriction and transparent denominator reporting over aggressive imputation across noncomparable eras [38,39]. Quantitative model-selection statistics, cohort accounting, operational definitions, follow-up availability, and sensitivity analyses are provided in Supplementary Tables S1–S5.

### 2.7. Software, Reproducibility, and Ethics/Data Governance

Data management and statistical analyses were conducted using scripted workflows to preserve reproducibility. All derived variables were generated from the raw public-use files using prespecified recoding rules verified against the SCIMS data dictionary, quick reference guide, and variable list. Variable availability by program era was checked before each analysis step, and model-specific denominators were reviewed against the intended analytic restrictions. Analysis code is available from the corresponding author upon reasonable request, subject to SCIMS public-use data-use restrictions.

The study used publicly available de-identified data and involved no direct contact with participants. The original SCIMS data were collected under center-level protocols and consent procedures established by the SCIMS program. Because the present study analyzed only de-identified public-use files, additional participant consent was not required. Under many local institutional policies, this type of secondary analysis is considered exempt from further ethics review or not to constitute human subject research. Final governance determinations were left to the authors’ local institutional requirements.

Data management and statistical analyses were conducted using Python version 3.13.5 (Python Software Foundation, Wilmington, DE, USA), with pandas version 2.2.3, NumPy version 2.3.5, SciPy version 1.17.0, statsmodels version 0.14.6, and matplotlib version 3.10.8. Visual and graphical refinement of the figures was assisted by ChatGPT, GPT-5.5 Thinking, latest available version at the time of manuscript preparation, OpenAI, San Francisco, CA, USA. All figure content, numerical values, manuscript text, and final visual outputs were critically reviewed, verified, and approved by the authors, who take full responsibility for the final content.

## 3. Results

The SCIMS 2021ARPublic release contained 35,675 Form I records, 131,217 Form II records, and 35,675 Record Status records, corresponding to 32,541 unique persons with at least one Form II entry. Of the Form II observations, 58,377 were coded as lost to follow-up and were not eligible for outcome ascertainment, leaving 72,840 non-lost interviews. At the person level, 29,310 individuals had at least one non-lost Form II interview. Among these, 28,745 contributed at least one non-missing rehospitalization indicator and entered the profile derivation cohort, whereas 565 were excluded because all four prespecified profile indicators were missing. Clinical-correlate analyses were restricted to 10,407 participants with the earliest eligible non-lost interview in 2016 or later and complete core correlate data. Participation analyses used domain-specific CHART samples ranging from 20,766 to 20,949 observations.

Baseline and follow-up characteristics by rehospitalization burden profile are summarized in Table 1. The profile cohort was predominantly male, with a mean age at injury of 34.5 years. Demographic differences across profiles were smaller than differences in follow-up intensity. The low and early/prolonged profiles each had a median of two non-lost interviews and a median maximum observed BYear of 5. The frequent/prolonged profile had a median of three non-lost interviews and a maximum observed BYear of 15, while the high recurrent/frequent/prolonged profile had a median of three non-lost interviews and a maximum observed BYear of 10. These differences support explicit caution that ever-positive indicators may be partly shaped by observation opportunity.

Quantitative model selection supported the four-profile solution (Supplementary Table S1). This solution had the lowest AIC and BIC and entropy of 0.709; five- and six-profile models did not improve penalized fit and produced smaller, less stable classes. Entropy was moderate (0.709), and average posterior probabilities for some profiles were below ideal levels; therefore, modal profile assignment should be interpreted with non-trivial classification uncertainty, particularly for individual-level classification. Class-defining conditional response probabilities and profile sizes are shown in Table 2. Missingness was highest for the recurrent indicator, which was unavailable for 11,139 participants (38.8%) with fewer than two informative waves, and for the early indicator, which was missing for 2607 participants (9.1%). The low rehospitalization burden profile included 17,180 participants (59.8%) and had low conditional probabilities across all indicators. The early/prolonged profile included 5445 participants (18.9%) and was characterized by high early rehospitalization probability and moderate prolonged-stay probability. The frequent/prolonged profile included 2213 participants (7.7%) and showed high probabilities of frequent and prolonged rehospitalization. The high recurrent/frequent/prolonged burden profile included 3907 participants (13.6%) and showed uniformly high probabilities across recurrence, frequency, duration, and early rehospitalization.

**Figure 2:**
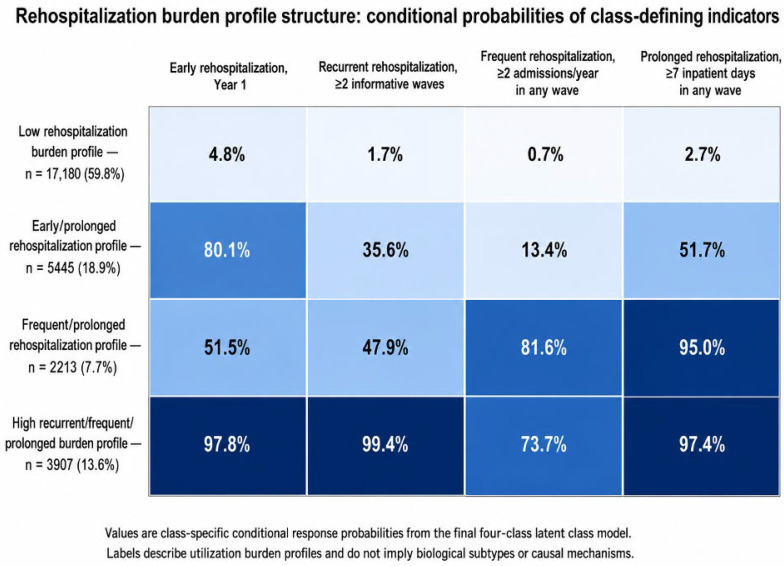
Rehospitalization burden profile structure: conditional probabilities of class-defining indicators. Values are class-specific conditional response probabilities from the final four-profile latent class model.

The probability heatmap in Figure 2 illustrates the internal structure of the four burden profiles. The low profile was consistently low across indicators. The early/prolonged profile was dominated by Year 1 rehospitalization and prolonged inpatient days, whereas the frequent/prolonged profile was dominated by within-wave admission frequency and prolonged inpatient days. The high recurrent/frequent/prolonged profile showed high conditional probabilities across all four indicators. Thus, the retained solution distinguishes observed timing, recurrence, within-wave frequency, and inpatient duration without implying biological subtypes or causal pathways.

Adjusted associations of candidate clinical correlates with profile membership are reported in Table 3. Using the low rehospitalization burden profile as the reference, UTI and pressure ulcer history showed the most consistent associations across burdened profiles. Any UTI was associated with the early/prolonged profile (OR 1.50, 95% CI 1.34–1.68), frequent/prolonged profile (OR 1.48, 95% CI 1.27–1.73), and high recurrent/frequent/prolonged profile (OR 2.31, 95% CI 2.00–2.67). Any pressure ulcer showed a stronger gradient, with ORs of 1.67 (95% CI 1.48–1.89), 2.54 (95% CI 2.17–2.97), and 2.74 (95% CI 2.40–3.14), respectively. Severe pain was most clearly associated with the frequent/prolonged and high recurrent/frequent/prolonged profiles. Clinically significant depressive symptoms showed smaller and less precise associations, while frequent sleep problems were most evident for the high recurrent/frequent/prolonged profile. These estimates are descriptive because the clinical-correlate interview may have preceded, overlapped with, or followed rehospitalization events contributing to profile assignment.

**Figure 3:**
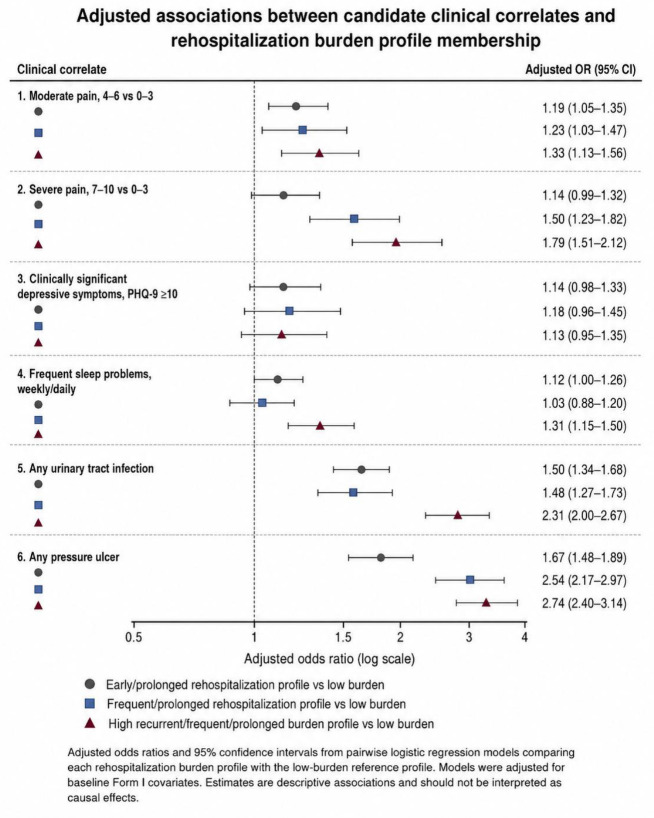
Adjusted associations between candidate clinical correlates and rehospitalization burden profile membership. Adjusted odds ratios and 95% confidence intervals are from pairwise logistic regression models comparing each burden profile with the low-burden reference profile. Estimates are descriptive associations and should not be interpreted as causal effects.

The adjusted associations are summarized visually in Figure 3. Unlike the previous tabular display, the revised forest plot shows effect sizes and 95% confidence intervals for all candidate clinical correlates, including UTI and pressure ulcer history. The visualization highlights the largest and most reproducible contrasts for UTI, pressure ulcer, severe pain, and frequent sleep problems in the heavier-burden profiles.

Adjusted participation differences by profile are shown in Table 4. All burden profiles had lower CHART scores than the low-burden reference profile, but the magnitude differed by domain and profile. The early/prolonged profile showed potentially meaningful deficits in physical independence, mobility, and occupation, with a smaller social-integration difference. The frequent/prolonged profile showed moderate deficits in physical independence and social integration, and large deficits in mobility and occupation. The high recurrent/frequent/prolonged profile showed large deficits in physical independence, mobility, and occupation, and a moderate deficit in social integration. Domain-specific analytic denominators were 20,949 for physical independence, 20,895 for mobility, 20,854 for occupation, and 20,766 for social integration.

The same gradient in participation burden was evident across adjusted participation models. The largest adjusted differences were concentrated in mobility and occupation, particularly for the frequent/prolonged and high recurrent/frequent/prolonged profiles. Physical independence followed a similar pattern, while social integration showed smaller but consistent differences. Timing-adjusted sensitivity models that added CHART measurement BYear, number of non-lost interviews, and maximum observed BYear produced similar direction and magnitude, supporting the robustness of the participation pattern while not eliminating residual aging or measurement-time confounding.

Descriptive rehospitalization reasons, expanded adjusted regression output, classification-quality summaries, incremental value comparisons, cohort fragmentation analyses, CHART timing-adjusted sensitivity models, and local-independence diagnostics are provided in Supplementary Tables S6–S12, respectively. Incremental value comparisons showed that cumulative rehospitalization count/day plus exposure models explained much of the CHART variation; adding profile membership provided limited additional explanatory information. Therefore, the profile framework should be viewed primarily as a descriptive and communicative summary of burden heterogeneity rather than as a replacement for simple count/day summaries when prediction is the primary objective.

## 4. Discussion

This study identified four descriptive rehospitalization burden profiles after traumatic SCI using clinically interpretable indicators derived from the SCIMS public-use dataset. The retained profiles captured heterogeneity in Year 1 occurrence, recurrence across informative waves, within-wave admission frequency, and prolonged inpatient days. The four-profile solution was supported by quantitative model-selection metrics and avoided the small, less stable additional partitions produced by more complex candidate solutions. These findings show that binary rehospitalization definitions can obscure important observed utilization patterns, while also emphasizing that the profiles are descriptive burden summaries rather than biological phenotypes or causal subtypes.

The follow-up exposure issue is central to interpretation. Recurrent, frequent, and prolonged indicators were defined as ever-positive across observed non-lost waves, so individuals with more interviews had a greater opportunity to meet burden thresholds. Sensitivity analyses restricted to participants with at least two or three informative waves retained broadly interpretable four-profile structures, but they cannot fully remove detection bias because the public-use dataset is wave-based and lacks continuous person-time. Local-independence diagnostics also indicated residual conditional dependence for pairs involving recurrence. These findings support using the profiles as pragmatic utilization summaries, not as definitive latent disease states. The incremental value analyses further indicate that simple cumulative count/day plus exposure measures remain strong explanatory summaries for CHART outcomes.

The clinical-correlate models help describe the profiles, but should not be interpreted causally. UTI and pressure ulcer history showed the most consistent associations with burdened profiles, especially the frequent/prolonged and high recurrent/frequent/prolonged profiles. This pattern is clinically coherent with SCI literature emphasizing multifactorial urological and skin complications [40,41,42,43,44,45,46,47]. However, UTI and pressure ulcers can also be reasons for rehospitalization, and the clinical-correlate interview may overlap with or follow events used to assign profiles. The observed associations may therefore reflect antecedents, consequences, admission reasons, shared clinical complexity, or some combination of these processes rather than independent causal predictors.

Pressure ulcer history showed the clearest gradient across profiles. This is plausible because pressure injuries often coexist with reduced mobility, skin risk, recurrent admissions, and longer hospital stays when advanced care is required. Newer work has pointed to metabolic, tissue, and body-composition factors that may amplify pressure injury risk in chronic SCI [46,47]. The present data cannot identify mechanisms, but the strong association between pressure ulcer history and heavier burden profiles supports the clinical relevance of skin surveillance as a hypothesis for future prospective testing.

Pain severity, depressive symptoms, and sleep problems add another descriptive layer. Severe pain was more strongly associated with the frequent/prolonged and high recurrent/frequent/prolonged profiles than moderate pain, while depressive symptoms showed smaller and less precise associations. Frequent sleep problems were most evident for the high recurrent/frequent/prolonged profile. These patterns are consistent with evidence that pain, psychological distress, and self-management challenges can cluster after SCI [48,49]. They should be read as markers of overall burden and potential care complexity rather than proof that symptom burden causes rehospitalization profile membership.

The CHART findings reinforce the rehabilitation relevance of the burden profiles. Compared with the low-burden profile, the heavier profiles showed lower participation scores, especially in mobility and occupation. Differences of 10 or more points were observed for mobility and occupation in the frequent/prolonged and high recurrent/frequent/prolonged profiles and can be regarded as large on the 0–100 scale, although domain-specific MCIDs for this registry context are not established. Timing-adjusted sensitivity models supported the same pattern. Nonetheless, because CHART outcomes were taken from the most recent valid non-lost interview, residual confounding by elapsed postinjury time, aging, and follow-up intensity remains possible.

Clinically, these profiles may be useful for communicating heterogeneous observed burden and for generating hypotheses about follow-up design. They suggest that early/prolonged, frequent/prolonged, and high recurrent/frequent/prolonged utilization patterns may have different rehabilitation implications. However, the present study does not test a profile-guided intervention. Structured follow-up, telehealth-enabled surveillance, bladder and skin prevention pathways, and transition-focused self-management support should therefore be framed as plausible future applications requiring external validation and prospective evaluation rather than as demonstrated consequences of the current analysis [50,51,52,53,54,55,56,57,58].

This study has several strengths. It used a large, nationally recognized public-use SCI dataset, applied a transparent profile derivation strategy, reported quantitative model-selection metrics, and linked burden profiles to both clinical correlates and participation outcomes. The revised analyses also report follow-up exposure, classification quality, local-independence diagnostics, sensitivity analyses, cohort fragmentation, and incremental value comparisons against simpler count/day formulations. Baseline age and neurological characteristics were included as adjustment variables, addressing the possibility that profile associations were confounded by major Form I clinical features.

Several limitations deserve careful consideration. The public-use dataset does not support granular event reconstruction, exact admission dates, or continuous time-to-event modeling. Follow-up is wave-based, and ever-positive indicators are sensitive to observation opportunity. Modal profile assignment ignores some classification uncertainty, and full bias-adjusted three-step methods were not implemented. Residual local dependence was present for recurrence-related indicator pairs. The magnitude of these bivariate residuals, particularly for recurrence-related pairs, further supports interpreting the retained profiles as pragmatic utilization summaries rather than well-separated latent constructs. The moderate entropy and lower average posterior probabilities for some profiles further reinforce this non-trivial classification uncertainty. Clinical correlates may have preceded, overlapped with, or followed rehospitalization events, and UTI or pressure ulcer variables may partly reflect the reasons for admission. The 2016+ clinical-correlate sample and CHART samples were smaller and not perfectly representative of the full profile cohort. Residual confounding by outpatient access, caregiver resources, bladder management details, prevention practices, aging, and measurement timing is likely. These constraints argue against causal interpretation and support viewing the profiles as descriptive, hypothesis-generating utilization summaries.

Future work should test whether similar burden profiles can be reproduced in other SCI cohorts and whether they remain stable under alternative indicator definitions, rate-based formulations, or dynamic longitudinal models. Studies with exact admission dates, richer outpatient data, and prospectively measured complications could better separate antecedents from consequences and quantify person-time at risk. Intervention studies should evaluate whether individuals matching heavier-burden profiles benefit from structured follow-up, enhanced skin and bladder surveillance, or transition-focused self-management support. Aging with SCI is another important next step because long-term rehabilitation and outpatient care must adapt to accumulating secondary conditions over time [59].

## 5. Conclusions

This secondary analysis of the SCIMS public-use dataset identified four descriptive rehospitalization burden profiles after traumatic SCI that captured observed heterogeneity in timing, recurrence, frequency, and duration of inpatient burden. The profiles were associated with clinical correlates and participation deficits, particularly UTI, pressure ulcer history, severe pain, mobility, and occupation. These associations are descriptive and may reflect overlapping complications, admission reasons, follow-up opportunities, or shared clinical complexity rather than causal pathways. The profile framework complements, but does not replace, simple count/day summaries. It may help communicate heterogeneous utilization burden and generate hypotheses for targeted follow-up strategies, but prospective validation is required before profile-informed risk stratification or care pathways can be recommended.

## Figures and Tables

**Table 1:** Baseline characteristics overall and by rehospitalization burden profile. Baseline characteristics are from SCIMS Form I at discharge from the index system hospitalization. The cohort is restricted to participants contributing at least one non-missing rehospitalization indicator to the latent class analysis (*N* = 28,745). Profile membership is based on modal assignment from the primary four-profile latent class model. Values are n (%) unless otherwise indicated; percentages are column percentages. Neurological level region at discharge was derived from the discharge neurological level. Follow-up summaries use all non-lost Form II interviews (BFolUpCt! = 5).

Characteristic	Overall	Low Rehospitalization Burden Profile	Early/Prolonged Rehospitalization Profile	Frequent/Prolonged Rehospitalization Profile	High Recurrent/ Frequent/Prolonged Burden Profile
Age at injury, mean (SD)	34.5 (16.3)	34.5 (16.4)	35.5 (17.0)	32.2 (14.7)	34.6 (15.9)
Sex: Male	23,063 (80.2%)	13,787 (80.3%)	4386 (80.6%)	1755 (79.3%)	3135 (80.2%)
Sex: Female	5678 (19.8%)	3390 (19.7%)	1059 (19.4%)	458 (20.7%)	771 (19.7%)
Sex: Other/Unknown	4 (0.0%)	3 (0.0%)	0 (0.0%)	0 (0.0%)	1 (0.0%)
Race: White	19,738 (68.7%)	11,560 (67.3%)	3802 (69.8%)	1613 (72.9%)	2763 (70.7%)
Race: Black	6339 (22.1%)	3830 (22.3%)	1188 (21.8%)	438 (19.8%)	883 (22.6%)
Race: Other	1344 (4.7%)	898 (5.2%)	232 (4.3%)	83 (3.8%)	131 (3.4%)
Race: Unknown	1324 (4.6%)	892 (5.2%)	223 (4.1%)	79 (3.6%)	130 (3.3%)
Ethnicity: Not Hispanic/Latino	25,749 (89.6%)	15,213 (88.6%)	4906 (90.1%)	2036 (92.0%)	3594 (92.0%)
Ethnicity: Hispanic/Latino	2793 (9.7%)	1833 (10.7%)	502 (9.2%)	163 (7.4%)	295 (7.6%)
Ethnicity: Unknown	203 (0.7%)	134 (0.8%)	37 (0.7%)	14 (0.6%)	18 (0.5%)
Neurological level region at discharge: Cervical	14,667 (51.0%)	8587 (50.0%)	2856 (52.5%)	1132 (51.2%)	2092 (53.5%)
Neurological level region at discharge: Thoracic	9817 (34.2%)	5661 (33.0%)	1875 (34.4%)	873 (39.4%)	1408 (36.0%)
Neurological level region at discharge: Lumbar	2720 (9.5%)	1952 (11.4%)	409 (7.5%)	130 (5.9%)	229 (5.9%)
Neurological level region at discharge: Sacral	86 (0.3%)	65 (0.4%)	16 (0.3%)	3 (0.1%)	2 (0.1%)
Neurological level region at discharge: Unknown/Other	1455 (5.1%)	915 (5.3%)	289 (5.3%)	75 (3.4%)	176 (4.5%)
Number of observed follow-up interviews (non-lost), median (IQR)	2 (1–3)	2 (1–3)	2 (1–3)	3 (2–5)	3 (1–4)
Maximum post-injury year observed, median (IQR)	5 (1–15)	5 (1–15)	5 (1–15)	15 (5–25)	10 (1–20)

Abbreviations: SCI, spinal cord injury; SCIMS, Spinal Cord Injury Model Systems; IQR, interquartile range; SD, standard deviation.

**Table 2:** Class-defining indicators and burden profile sizes. Indicators were derived from non-lost Form II interviews (BFolUpCt! = 5). Early rehospitalization was defined as any rehospitalization reported at the standard Year 1 interview. Recurrent rehospitalization was defined as rehospitalization in ≥2 informative waves. Frequent rehospitalization was defined as ≥2 rehospitalizations in any wave. Prolonged rehospitalization was defined as ≥7 inpatient days in any wave. The latent class model was estimated using full-information maximum likelihood under local independence as a working assumption; percentages are class-specific conditional response probabilities. Class sizes reflect modal assignment and sum to the profile cohort (*N* = 28,745). Profile labels are descriptive and do not imply causal mechanisms.

Profile	Class Size, n (%)	Early (Year 1)	Recurrent (≥2 waves)	Frequent (≥2/year)	Prolonged (≥7 days)
Low rehospitalization burden profile	17,180 (59.8%)	4.8%	1.7%	0.7%	2.7%
Early/prolonged rehospitalization profile	5445 (18.9%)	80.1%	35.6%	13.4%	51.7%
Frequent/prolonged rehospitalization profile	2213 (7.7%)	51.5%	47.9%	81.6%	95.0%
High recurrent/frequent/prolonged burden profile	3907 (13.6%)	97.8%	99.4%	73.7%	97.4%

Abbreviations: LCA, latent class analysis; FIML, full-information maximum likelihood.

**Table 3:** Adjusted associations of candidate clinical correlates with rehospitalization burden profile membership (each profile vs low rehospitalization burden). Values are adjusted odds ratios (95% CI) from pairwise multivariable logistic regression models comparing each burden profile with the low rehospitalization burden profile (reference). One observation per participant was used (*N* = 10,407), defined as the earliest non-lost Form II interview in 2016 or later with complete core clinical-correlate data. Models were adjusted for baseline Form I covariates: age at injury, sex, race, Hispanic origin, broad traumatic etiology, discharge neurological level region, and discharge AIS grade. Discharge neurological category was evaluated but not retained due to collinearity/instability with neurological level and AIS. Associations are descriptive and should not be interpreted as causal effects.

Clinical Correlate (Reference)	Early/Prolonged Rehospitalization Profile	Frequent/Prolonged Rehospitalization Profile	High Recurrent/ Frequent/Prolonged Burden Profile
Moderate pain (4–6) vs. 0–3	1.19 (1.05–1.35)	1.23 (1.03–1.47)	1.33 (1.13–1.56)
Severe pain (7–10) vs. 0–3	1.14 (0.99–1.32)	1.50 (1.23–1.82)	1.79 (1.51–2.12)
Clinically significant depressive symptoms (PHQ-9 ≥10)	1.14 (0.98–1.33)	1.18 (0.96–1.45)	1.13 (0.95–1.35)
Frequent sleep problems (weekly/daily)	1.12 (1.00–1.26)	1.03 (0.88–1.20)	1.31 (1.15–1.50)
Any urinary tract infection	1.50 (1.34–1.68)	1.48 (1.27–1.73)	2.31 (2.00–2.67)
Any pressure ulcer	1.67 (1.48–1.89)	2.54 (2.17–2.97)	2.74 (2.40–3.14)

Abbreviations: AIS, American Spinal Injury Association Impairment Scale; CI, confidence interval; OR, odds ratio; PHQ-9, Patient Health Questionnaire-9; UTI, urinary tract infection.

**Table 4:** Adjusted differences in CHART participation outcomes by rehospitalization burden profile (each profile vs. low rehospitalization burden). Values are adjusted mean differences (beta; 95% CI) from linear regression models with robust standard errors comparing each burden profile with the low rehospitalization burden profile (reference). Outcomes are CHART domain totals (0–100); higher scores indicate better participation. Each domain was modeled using the most recent non-lost Form II interview with a non-missing score for that specific domain. Models used the same baseline adjustment set as Table 3. *N* denotes the final domain-specific analysis sample after applying outcome and covariate availability criteria. Associations are descriptive and should not be interpreted as causal effects.

Outcome (Higher Is Better)	Early/Prolonged Rehospitalization Profile	Frequent/Prolonged Rehospitalization Profile	High Recurrent/ Frequent/Prolonged Burden Profile	N (Analysis)
CHART Physical Independence	−5.6 (−6.7 to −4.4)	−7.5 (−9.1 to −6.0)	−11.1 (−12.4 to −9.8)	20,949
CHART Mobility	−5.4 (−6.4 to −4.5)	−10.9 (−12.3 to −9.5)	−13.8 (−15.0 to −12.7)	20,895
CHART Occupation	−6.1 (−7.4 to −4.8)	−10.5 (−12.3 to −8.8)	−13.3 (−14.7 to −11.9)	20,854
CHART Social Integration	−2.1 (−3.0 to −1.2)	−5.0 (−6.2 to −3.7)	−6.1 (−7.2 to −5.0)	20,766

Abbreviations: CHART, Craig Handicap Assessment and Reporting Technique; CI, confidence interval.

## Data Availability

The data supporting the findings of this study are available as part of the SCIMS Public Use Dataset (2021ARPublic) from the National Spinal Cord Injury Statistical Center. De-identified data can be accessed by qualified investigators in accordance with the applicable data use agreement. The authors are not permitted to redistribute the dataset directly.

## Supplementary Materials

The following supporting information can be downloaded at: https://www.mdpi.com/article/10.3390/jcm15134890/s1, Table S1: Quantitative latent class model-selection metrics; Table S2: Cohort assembly and analytic denominators; Table S3: Operational definitions, recoding rules, and missing-data conventions; Table S4: Follow-up availability and observation intensity by profile; Table S5: Sensitivity analyses with observed findings; Table S6: Descriptive rehospitalization reasons overall and by profile; Table S7: Expanded adjusted regression output for focal predictors and CHART outcomes; Table S8: Classification-quality reporting for the primary four-profile solution; Table S9: Incremental value comparisons versus simple count/day formulations; Table S10: (A) Cohort fragmentation and standardized differences; (B). Standardized differences for analytic subsets relative to LCA/profile cohort; Table S11: CHART measurement-time adjusted sensitivity models; Table S12: Local-independence diagnostics.
