# Supplementary material for: Rehospitalization Burden Profiles After Traumatic Spinal Cord Injury: A Data-Driven Latent Class Analysis of the SCIMS Public-Use Database
Source: J Clin Med. 2026 Jun 23;15(13):4890. doi: 10.3390/jcm15134890 (PMC13361992; doi:10.3390/jcm15134890)
Supplement: Supplementary file 1 [file jcm-15-04890-s001.zip › jcm-4308279-supplementary.pdf]

Supplementary Material

Rehospitalization burden profiles after traumatic spinal cord injury: a data-driven latent class analysis of the SCIMS public-use database

Supplementary Table S1. Quantitative latent class model-selection metrics.

Candidate models with two through six classes were fitted to the four rehospitalization indicators. AIC and BIC favored the four-class solution; the five- and six-class models did not provide sufficient improvement to justify the additional complexity.

No. classes	Log-likelihood	Parameters	AIC	BIC	Entropy	Smallest modal class, n (%)	Average posterior probability range	Retained primary?
2	-46,916.632	9	93,851.3	93,925.7	0.850	9,510 (33.1)	0.961-0.967	No
3	-46,682.296	14	93,392.6	93,508.3	0.794	3,541 (12.3)	0.599-0.958	No
4	-46,549.158	19	93,136.3	93,293.4	0.709	2,213 (7.7)	0.603-0.935	Yes
5	-46,549.151	24	93,146.3	93,344.7	0.667	603 (2.1)	0.674-0.897	No
6	-46,549.143	29	93,156.3	93,396.0	0.585	334 (1.2)	0.541-0.798	No

Supplementary Table S2. Cohort assembly and analytic denominators.

Stepwise accounting of the SCIMS 2021ARPublic cohort and the denominators used in the main manuscript.

Cohort step	N	Definition
SCIMS 2021ARPublic Form I records	35,675	Baseline/public-use Form I file
SCIMS 2021ARPublic Form II records	131,217	All Form II follow-up records in public-use release
SCIMS 2021ARPublic Record Status records	35,675	Vital/status file for cohort accounting
Unique persons in Form II	32,541	Distinct UniID values in Form II
Form II interviews lost to follow-up (BFolUpCt = 5)	58,377	Not observed for outcome ascertainment
Eligible non-lost Form II interviews	72,840	All remaining non-lost Form II interviews
Unique persons with >=1 non-lost Form II interview	29,310	Distinct participants contributing eligible follow-up
Participants with >=1 nonmissing rehospitalization indicator (LCA/profile cohort)	28,745	Primary profile analysis sample
Participants with all 4 rehospitalization indicators missing	565	Excluded from profile derivation
Clinical-correlate analysis sample	10,407	Earliest non-lost 2016+ interview with complete core clinical-correlate data
CHART Physical Independence model N	20,949	Updated domain-specific analysis sample
CHART Mobility model N	20,895	Updated domain-specific analysis sample
CHART Occupation model N	20,854	Updated domain-specific analysis sample
CHART Social Integration model N	20,766	Updated domain-specific analysis sample

Supplementary Table S3. Operational definitions, recoding rules, and missing-data conventions.

Variable-level operational choices used in the revised analyses.

Variable / construct	Source	Meaning	Valid / special codes	Analytic recode	Missing-data convention
BFolUpCt	Form II	Category of follow-up care	5 = lost to follow-up	Lost interviews excluded from outcome ascertainment	Blank by design for lost interviews
BRhspNbr	Form II	No. rehospitalizations in last 12 months	0 none; 1-6 exact; 7 >6; 8 rehospitalized, number unknown; 9 unknown	Any rehospitalization positive if 1-8; missing if 9	Code 8 missing for frequency thresholding
BRhspDaT	Form II	Total days rehospitalized in last 12 months	0 none; 1-887 valid; 888 days unknown; 999 unknown	Prolonged positive if >=7 days; missing if 888/999	Sensitivity repeated at >=14 days
Early rehospitalization	Derived	Any rehospitalization at standard Year 1 interview	BYear=1 and BRhspNbr >0	Positive if BRhspNbr 1-8; negative if 0; missing if Year 1 unavailable or BRhspNbr=9	Wave-based indicator
Recurrent rehospitalization	Derived	Rehospitalization in >=2 informative non-lost waves	Informative wave requires BRhspNbr not 9	Missing if <2 informative waves	Prevents sparse follow-up from being forced into nonrecurrent category
Frequent rehospitalization	Derived	>=2 admissions in any observed wave	Positive if BRhspNbr 2-7	Missing if only 8/9 or structural missing for thresholding	Sensitivity repeated at >=3 admissions
Prolonged rehospitalization	Derived	>=7 total inpatient days in any observed wave	Positive if BRhspDaT >=7	Missing if only 888/999 or structural missing	Sensitivity repeated at >=14 days
BPainSev	Form II	Usual pain over past 4 weeks (0-10)	99 unknown	Categorized 0-3, 4-6, 7-10	Core clinical correlate
BBPHQSDS	Form II	PHQ-9 total score (0-27)	99 unknown/declined/interview not done/age <18	Clinically significant symptoms >=10	Core clinical correlate
BSleep	Form II	Sleep problems frequency	0 never/<monthly; 1 monthly; 2 weekly; 3 daily/almost daily; 9 unknown	Frequent sleep problems yes for 2 or 3	Available for harmonized modern-era analyses
BUTI	Form II	UTI requiring antibiotics	0 none; 1/3/5/6 any UTI; 7 declined/don't know; 9 unknown	Any UTI yes for 1/3/5/6; missing for 7/9	Added in 2011
BPrUlcer	Form II	Pressure ulcer in last 12 months	0 no; 1 yes; 7 declined/don't know; 9 unknown	Any pressure ulcer yes for 1; missing for 7/9	Added in 2011
BCHPITot/BCHMbTot/BCHOpTot/BCHSocIn	Form II	CHART domain scores	0-100 valid; 999 unknown/interview not done	Most recent valid non-lost interview per domain	999 treated as missing

Supplementary Table S4. Follow-up availability and observation intensity by profile.

Profile-specific follow-up intensity summaries aligned with the revised primary four-profile solution.

Profile	Class size, n (%)	Average posterior probability	Non-lost follow-up interviews, median [IQR]	Maximum observed post-injury year, median [IQR]
Low rehospitalization burden profile	17,180 (59.8%)	0.935	2 [1, 3]	5 [1, 15]
Early/prolonged rehospitalization profile	5,445 (18.9%)	0.686	2 [1, 3]	5 [1, 15]
Frequent/prolonged rehospitalization profile	2,213 (7.7%)	0.842	3 [2, 5]	15 [5, 25]
High recurrent/frequent/prolonged burden profile	3,907 (13.6%)	0.603	3 [1, 4]	10 [1, 20]

Supplementary Table S5. Sensitivity analyses with observed findings.

Sensitivity analyses assessing threshold choice, follow-up exposure, and classification uncertainty.

Sensitivity analysis	Operational definition	Analytic N	% of primary cohort retained	Observed impact	Stability metric / note
Primary four-profile solution	Primary thresholds: frequent >=2 admissions; prolonged >=7 days	28,745	100.0	Profiles: low 17,180; early/prolonged 5,445; frequent/prolonged 2,213; high recurrent/frequent/prolonged 3,907	Reference
Stricter burden thresholds	Frequent >=3 admissions; prolonged >=14 days	28,745	100.0	Four-class solution retained broad low, early/intermediate, high, and small frequent/prolonged structure	ARI vs primary = 0.791
>=2 informative non-lost waves	Restricted to participants with at least 2 informative waves	17,606	61.2	Four-class solution retained interpretable low and burdened structures	ARI vs primary among overlapping participants = 0.741
>=3 informative non-lost waves	Restricted to participants with at least 3 informative waves	10,521	36.6	Four-class solution retained interpretable low and burdened structures	ARI vs primary among overlapping participants = 0.798
Exclude max posterior probability <0.70	Retain only participants with maximum posterior probability >=0.70	21,035	73.2	Sensitivity threshold for uncertain assignment	Retained 21,035 participants
Exclude max posterior probability <0.80	Retain only participants with maximum posterior probability >=0.80	18,664	64.9	More stringent threshold for uncertain assignment	Retained 18,664 participants
Exclude BRhspNbr = 8 observations	Participants with any non-lost wave coded rehospitalized but number unknown	151	0.5 affected	Sensitivity targets unknown-count admissions in threshold models	Small affected count

Supplementary Table S6. Descriptive rehospitalization reasons overall and by profile.

Nonmissing reason positions BRhspRs1-BRhspRs8 were stacked to the event level among non-lost Form II interviews. Codes 88 and 99 were excluded.

Reason code	Primary reason for rehospitalization	Overall events, n (%)	Low rehospitalization burden profile	Early/prolonged rehospitalization profile	Frequent/prolonged rehospitalization profile	High recurrent/frequent/prolonged burden profile
1	Infectious and parasitic diseases, including AIDS	864 (4.4%)	96 (3.7%)	150 (4.0%)	225 (4.6%)	393 (4.8%)
2	Cancer	152 (0.8%)	23 (0.9%)	28 (0.7%)	38 (0.8%)	63 (0.8%)
3	Endocrine, nutritional and metabolic disorders	171 (0.9%)	33 (1.3%)	31 (0.8%)	27 (0.5%)	80 (1.0%)
4	Diseases of blood and blood-forming organs	358 (1.8%)	45 (1.7%)	65 (1.7%)	88 (1.8%)	160 (2.0%)
5	Mental disorders, including alcohol/drug-related problems	277 (1.4%)	32 (1.2%)	48 (1.3%)	83 (1.7%)	114 (1.4%)
6	Disease of nervous system and sense organs	376 (1.9%)	55 (2.1%)	92 (2.4%)	94 (1.9%)	135 (1.7%)
7	Diseases of the circulatory system	1085 (5.6%)	172 (6.6%)	246 (6.5%)	248 (5.0%)	419 (5.1%)
8	Diseases of the respiratory system	1452 (7.5%)	141 (5.4%)	288 (7.6%)	343 (7.0%)	680 (8.3%)
9	Diseases of the digestive system	1314 (6.7%)	241 (9.2%)	232 (6.1%)	326 (6.6%)	515 (6.3%)
10	Diseases of the	5400 (27.7%)	579 (22.2%)	1028 (27.1%)	1275 (25.9%)	2518 (30.9%)

	genitourinary system (including UTI)					
11	Childbirth / pregnancy complications	164 (0.8%)	63 (2.4%)	17 (0.4%)	45 (0.9%)	39 (0.5%)
12	Diseases of skin and subcutaneous tissue (including pressure ulcers)	3077 (15.8%)	350 (13.4%)	428 (11.3%)	981 (19.9%)	1318 (16.2%)
13	Diseases of musculoskeletal system and connective tissue	1124 (5.8%)	194 (7.4%)	264 (7.0%)	266 (5.4%)	400 (4.9%)
14	Congenital anomalies	11 (0.1%)	1 (0.0%)	3 (0.1%)	2 (0.0%)	5 (0.1%)
15	Symptoms and ill-defined conditions	409 (2.1%)	76 (2.9%)	109 (2.9%)	95 (1.9%)	129 (1.6%)
16	Injuries and poisoning / surgical or medical complications	946 (4.9%)	198 (7.6%)	226 (6.0%)	209 (4.2%)	313 (3.8%)
17	Other unclassified (e.g., baclofen pump)	1750 (9.0%)	288 (11.0%)	433 (11.4%)	393 (8.0%)	636 (7.8%)
18	Inpatient rehabilitation services only	553 (2.8%)	23 (0.9%)	108 (2.8%)	191 (3.9%)	231 (2.8%)

Supplementary Table S7. Expanded adjusted regression output for focal predictors and CHART outcomes.

Part A provides adjusted odds ratios for clinical correlate models. Part B provides adjusted beta coefficients for CHART participation models.

Comparison	Predictor / outcome	Effect metric	Estimate	95% CI low	95% CI high	Analytic N
Early/prolonged rehospitalization profile vs low	Moderate pain (4-6) vs 0-3	OR	1.193	1.051	1.355	8160
Early/prolonged rehospitalization profile vs low	Severe pain (7-10) vs 0-3	OR	1.142	0.988	1.319	8160
Early/prolonged rehospitalization profile vs low	Clinically significant depressive symptoms (PHQ-9 >=10)	OR	1.142	0.978	1.334	8160
Early/prolonged rehospitalization profile vs low	Frequent sleep problems (weekly/daily)	OR	1.121	0.999	1.257	8160
Early/prolonged rehospitalization profile vs low	Any urinary tract infection	OR	1.499	1.337	1.680	8160
Early/prolonged rehospitalization profile vs low	Any pressure ulcer	OR	1.674	1.480	1.893	8160
Frequent/prolonged rehospitalization profile vs low	Moderate pain (4-6) vs 0-3	OR	1.228	1.027	1.469	7199
Frequent/prolonged rehospitalization profile vs low	Severe pain (7-10) vs 0-3	OR	1.497	1.231	1.820	7199
Frequent/prolonged rehospitalization profile vs low	Clinically significant depressive symptoms (PHQ-9 >=10)	OR	1.183	0.963	1.453	7199
Frequent/prolonged rehospitalization profile vs low	Frequent sleep problems (weekly/daily)	OR	1.031	0.882	1.205	7199
Frequent/prolonged rehospitalization profile vs low	Any urinary tract infection	OR	1.482	1.266	1.735	7199
Frequent/prolonged rehospitalization profile vs low	Any pressure ulcer	OR	2.536	2.169	2.966	7199
High recurrent/frequent/prolonged burden profile vs low	Moderate pain (4-6) vs 0-3	OR	1.328	1.132	1.557	7644
High recurrent/frequent/prolonged burden profile vs low	Severe pain (7-10) vs 0-3	OR	1.789	1.509	2.120	7644
High recurrent/frequent/prolonged burden profile vs low	Clinically significant depressive symptoms (PHQ-9 >=10)	OR	1.135	0.951	1.355	7644
High recurrent/frequent/prolonged burden profile vs low	Frequent sleep problems (weekly/daily)	OR	1.312	1.145	1.504	7644
High recurrent/frequent/prolonged burden profile vs low	Any urinary tract infection	OR	2.308	1.995	2.669	7644
High	Any pressure ulcer	OR	2.742	2.396	3.138	7644

recurrent/frequent/prolonged burden profile vs low						
Early/prolonged rehospitalization profile vs low burden	CHART Physical Independence	beta	-5.553	-6.657	-4.449	20,949
Frequent/prolonged rehospitalization profile vs low burden	CHART Physical Independence	beta	-7.520	-9.059	-5.981	20,949
High recurrent/frequent/prolonged burden profile vs low burden	CHART Physical Independence	beta	-11.096	-12.435	-9.757	20,949
Early/prolonged rehospitalization profile vs low burden	CHART Mobility	beta	-5.417	-6.381	-4.453	20,895
Frequent/prolonged rehospitalization profile vs low burden	CHART Mobility	beta	-10.909	-12.323	-9.496	20,895
High recurrent/frequent/prolonged burden profile vs low burden	CHART Mobility	beta	-13.817	-14.965	-12.669	20,895
Early/prolonged rehospitalization profile vs low burden	CHART Occupation	beta	-6.097	-7.380	-4.813	20,854
Frequent/prolonged rehospitalization profile vs low burden	CHART Occupation	beta	-10.543	-12.272	-8.814	20,854
High recurrent/frequent/prolonged burden profile vs low burden	CHART Occupation	beta	-13.337	-14.741	-11.933	20,854
Early/prolonged rehospitalization profile vs low burden	CHART Social Integration	beta	-2.095	-3.004	-1.186	20,766
Frequent/prolonged rehospitalization profile vs low burden	CHART Social Integration	beta	-4.958	-6.220	-3.697	20,766
High recurrent/frequent/prolonged burden profile vs low burden	CHART Social Integration	beta	-6.133	-7.221	-5.044	20,766

Supplementary Table S8. Classification-quality reporting for the primary four-profile solution.

Classification quality and posterior-probability sensitivity summaries.

Item	Reporting statement
Selected model	Four-profile latent class solution
Entropy	0.709
Modal class sizes	Low: 17,180 (59.8%); early/prolonged: 5,445 (18.9%); frequent/prolonged: 2,213 (7.7%); high recurrent/frequent/prolonged: 3,907 (13.6%)
Average posterior probability, low burden	0.935
Average posterior probability, early/prolonged	0.686
Average posterior probability, frequent/prolonged	0.842
Average posterior probability, high recurrent/frequent/prolonged	0.603
Participants with maximum posterior probability >=0.70	21,035 (73.2%)
Participants with maximum posterior probability >=0.80	18,664 (64.9%)
Interpretive note	Modal assignment was used for transparent downstream reporting, supported by posterior-probability sensitivity analyses. Profile labels are descriptive and not causal.

Supplementary Table S9. Incremental value comparisons versus simple count/day formulations.

Nested models compare baseline covariates, cumulative rehospitalization count and inpatient days, follow-up exposure indicators, and profile membership for continuous CHART outcomes.

Outcome	Model	N	AIC	BIC	Adj. R2	RMSE	Delta AIC vs Model B	Delta BIC vs Model B	Delta adjusted R2 vs Model B
CHART Mobility	A baseline	20,895	195,833.5	196,016.3	0.130	26.209	1,267.8	1,236.0	-0.051
CHART Mobility	B count/day + exposure	20,895	194,565.7	194,780.3	0.181	25.421	0.000	0.000	0.000
CHART Mobility	C profile	20,895	195,033.5	195,240.1	0.162	25.708	467.826	459.879	-0.019
CHART Mobility	D count/day + exposure + profile	20,895	194,553.1	194,791.6	0.182	25.409	-12.551	11.290	0.001
CHART Occupation	A baseline	20,854	207,376.7	207,559.5	0.154	34.885	687.916	656.135	-0.028
CHART Occupation	B count/day + exposure	20,854	206,688.8	206,903.3	0.181	34.308	0.000	0.000	0.000
CHART Occupation	C profile	20,854	206,953.8	207,160.4	0.171	34.528	265.016	257.071	-0.011
CHART Occupation	D count/day + exposure + profile	20,854	206,690.4	206,928.8	0.181	34.304	1.592	25.427	0.000
CHART Physical Independence	A baseline	20,949	201,789.8	201,972.7	0.198	29.850	706.731	674.932	-0.027
CHART Physical Independence	B count/day + exposure	20,949	201,083.1	201,297.7	0.225	29.345	0.000	0.000	0.000
CHART Physical Independence	C profile	20,949	201,405.4	201,612.1	0.213	29.573	322.341	314.391	-0.012
CHART Physical Independence	D count/day + exposure + profile	20,949	201,084.2	201,322.7	0.225	29.342	1.145	24.995	0.000
CHART Social Integration	A baseline	20,766	191,829.9	192,012.5	0.036	24.503	300.155	268.390	-0.014
CHART Social Integration	B count/day + exposure	20,766	191,529.7	191,744.1	0.050	24.322	0.000	0.000	0.000
CHART Social Integration	C profile	20,766	191,657.6	191,864.0	0.044	24.398	127.827	119.886	-0.006
CHART Social Integration	D count/day + exposure + profile	20,766	191,530.7	191,768.9	0.050	24.319	0.969	24.792	0.000

Supplementary Table S10. A. Cohort fragmentation and standardized differences.

The first block summarizes analytic samples. The second block reports standardized differences relative to the primary LCA/profile cohort.

Sample	N	Age at injury, mean (SD)	Male, n (%)	Cervical injury, n (%)	Non-lost interviews, median [IQR]	Maximum observed year, median [IQR]	White race, n (%)
Primary LCA/profile cohort	28,745	34.5 (16.3)	23,063 (80.2)	14,667 (51.0)	2 [1, 3]	5 [1, 15]	19,738 (68.7)
2016+ complete clinical-correlate sample	10,407	34.4 (16.0)	8,146 (78.3)	5,065 (48.7)	3 [2, 4]	10 [5, 25]	7,330 (70.4)
Any valid CHART participation sample	21,052	35.3 (16.4)	16,684 (79.3)	10,621 (50.5)	2 [1, 4]	10 [5, 20]	14,691 (69.8)

Supplementary Table S10. B. Standardized differences for analytic subsets relative to LCA/profile cohort.

Absolute SMD values around 0.10 are commonly considered small, but interpretation should consider context and sample size.

Comparison	Age at injury SMD	Male SMD	Cervical injury SMD	White race SMD	No. non-lost interviews SMD	Maximum observed year SMD
Clinical-correlate sample vs LCA cohort	-0.005	-0.048	-0.047	0.038	0.348	0.322
CHART sample vs LCA cohort	0.045	-0.024	-0.011	0.024	0.209	0.224

Supplementary Table S11. CHART measurement-time adjusted sensitivity models.

Models add CHART measurement BYear, number of non-lost interviews, and maximum observed BYear to the baseline adjustment set.

Outcome	Comparison	Adjusted beta	95% CI low	95% CI high	N
CHART Physical Independence	Early/prolonged rehospitalization profile vs low burden	-5.692	-6.791	-4.592	20,949
CHART Physical Independence	Frequent/prolonged rehospitalization profile vs low burden	-9.425	-10.978	-7.872	20,949
CHART Physical Independence	High recurrent/frequent/prolonged burden profile vs low burden	-12.286	-13.624	-10.948	20,949
CHART Mobility	Early/prolonged rehospitalization profile vs low burden	-5.349	-6.310	-4.389	20,895
CHART Mobility	Frequent/prolonged rehospitalization profile vs low burden	-10.418	-11.848	-8.988	20,895
CHART Mobility	High recurrent/frequent/prolonged burden profile vs low burden	-13.611	-14.765	-12.456	20,895
CHART Occupation	Early/prolonged rehospitalization profile vs low burden	-6.023	-7.302	-4.743	20,854
CHART Occupation	Frequent/prolonged rehospitalization profile vs low burden	-10.056	-11.807	-8.304	20,854
CHART Occupation	High recurrent/frequent/prolonged	-13.152	-14.566	-11.738	20,854

	burden profile vs low burden				
CHART Social Integration	Early/prolonged rehospitalization profile vs low burden	-2.028	-2.935	-1.121	20,766
CHART Social Integration	Frequent/prolonged rehospitalization profile vs low burden	-4.346	-5.625	-3.066	20,766
CHART Social Integration	High recurrent/frequent/prolonged burden profile vs low burden	-5.801	-6.902	-4.700	20,766

Supplementary Table S12. Local-independence diagnostics.

Bivariate residuals are reported for each pair of class-defining indicators. Values >3.84 suggest residual conditional dependence at an approximate 0.05 chi-square threshold with 1 degree of freedom.

Indicator pair	Observed N	Bivariate residual chi-square	Observed phi	Flag (>3.84)
Early (Year 1) vs Recurrent (>=2 waves)	16,077	195.254	0.473	Yes
Early (Year 1) vs Frequent (>=2/year)	26,100	0.137	0.391	No
Early (Year 1) vs Prolonged (>=7 days)	25,882	2.615	0.552	No
Recurrent (>=2 waves) vs Frequent (>=2/year)	17,606	290.286	0.469	Yes
Recurrent (>=2 waves) vs Prolonged (>=7 days)	17,597	382.815	0.532	Yes
Frequent (>=2/year) vs Prolonged (>=7 days)	28,456	1.713	0.601	No
